# Endocrowns: Indications, Preparation Techniques, and Material Selection

**DOI:** 10.7759/cureus.49947

**Published:** 2023-12-05

**Authors:** Dalal S AlDabeeb, Nouf S Alakeel, Raneem M Al jfshar, Thakra K Alkhalid

**Affiliations:** 1 Department of Restorative Dental Sciences, King Saud University, Riyadh, SAU; 2 College of Dentistry, King Saud University, Riyadh, SAU

**Keywords:** materials, cementation, contraindication, preparation technique, indications, endodontically treated tooth, endocrowns

## Abstract

Endodontic treatment is often necessary in the field of dentistry. As the tooth structure is lost during such treatment, the tooth may become weaker and lose some of its mechanical qualities. Endodontically treated posterior teeth require cuspal coverage because of their anatomical features. Endocrowns are regarded as a suitable choice for restoring teeth that have undergone endodontic treatment. These restorations are recommended when there is a substantial loss of tooth structure, restricted interocclusal space, or a short clinical crown. They are also contraindicated in case of severe loss of tooth structure where adhesion is not applicable. Endocrowns require a specific preparation design that is distinct from the conventional crown. They can be manufactured by two methods: heat pressing or computer-aided design/computer-aided manufacturing (CAD/CAM). Moreover, several materials have been used in fabricating endocrown restoration. Lithium disilicate glass-ceramic is the most recommended material as it possesses excellent mechanical properties and esthetic results with the ability to bond to tooth structure. In conclusion, several kinds of literature recommend using them for molars. Further research is needed to evaluate this technique for premolar and anterior teeth.

## Introduction and background

Endodontic treatment is common in dental practice [1]. However, it can weaken the tooth and reduce its mechanical properties due to the loss of tooth structure [1-3]. This loss of structure reduces the fracture resistance, affecting the tooth’s long-term survival rate [2,4]. Causes of tooth structure loss can include caries, trauma, access cavities, and canal preparation [2,3,5]. Additionally, endodontically treated teeth have reduced protection against mastication forces due to deterioration in the neurosensory feedback system after the removal of pulpal tissue [2,3]. This reduced feedback system increases the pressure threshold in the periodontium, leading to functional overload and decreased fracture resistance [5].

The success and longevity of endodontically treated teeth rely on treatment quality and proper coronal restoration to maintain the tooth’s function, form, and aesthetics [5,6]. It is recommended to proceed with the final tooth restoration once the root canal treatment meets technical standards and the tooth is symptom-free [6]. The best restoration method for endodontically treated teeth has been debated extensively in the literature [5,7]. However, significant advancements have been made in the past 20 years [6], and these advancements primarily emphasize the preservation of tooth structure [6].

The final restoration depends on the amount of remaining structure and the type of tooth, for example, whether it is anterior or posterior [6,8]. Anterior teeth with an adequate amount of tooth structure and a limited endodontic access cavity can be restored with a direct restoration without the need for complete tooth coverage with a crown [8].

Nevertheless, in dealing with posterior teeth, cuspal coverage is always required because of their anatomical features and the higher stress they endure [8]. Endodontically treated teeth with considerable loss of coronal tooth structure are usually treated with core buildup and crown [2,8]. If the remaining tooth structure is insufficient to keep the core in place, additional retentive mechanisms, such as a post, may be used [8]. The post, which can be either prefabricated or custom-made, retains the core [8]. However, studies have concluded that endodontically treated teeth with posts have comparable failure modes and similar fracture resistance to endodontically treated teeth without posts. This indicates posts may not be necessary [3].

Studies show that using intracanal retention can weaken the tooth, rendering it more susceptible to fractures [2,8]. Furthermore, applying posts increases the risk of perforations and complicates the potential for future endodontic re-treatment [2,3,8]. These factors, combined with the fact that restorative dentistry opposes the waste of dental tissue, make endocrowns a desirable alternative [3].

With the advancement of adhesive techniques and an increased focus on minimally invasive procedures, restorative alternatives such as endocrowns are now available to restore endodontically treated teeth [2,7]. Pissis developed the technique for endocrown restoration in 1995, but the term endocrown was coined by Bindl and Mormann in 1999 [9]. They described endocrowns as monolithic, single-piece restorations made of either full-composite or full-ceramic materials that partially or totally restore the coronal portion of an endodontically treated tooth [9]. These restorations rely on macromechanical retention, achieved by anchoring the restoration within the pulp chamber’s internal portion and to the cavity’s margin [7,9]. They also rely on micromechanical retention using adhesive cementation [7,9].

Compared to conventional post and core restorations, endocrowns offer an easier and more straightforward procedure [3,9]. They require less time and cost while providing enhanced aesthetic properties [3,9]. Additionally, the adhesive technique used in endocrowns prevents marginal leakage and minimizes the penetration of microorganisms from the crown toward the root, thereby contributing to the success of the endodontic treatment [2,10]. In addition, several studies have demonstrated that endocrowns exhibit a lower incidence of catastrophic failures, for example, those that require the extraction of the affected tooth [6,10]. They also provide better stress distribution and increased fracture resistance compared to conventional crowns [1,3,5,9-11].

## Review

Indications

Endocrown restoration is recommended for teeth with significant loss of tooth structure and limited interocclusal space, which can make it difficult to achieve an adequate material thickness for conventional crowns [3,7]. Furthermore, endocrown restoration is indicated for cases where it is difficult to apply an adequate ferrule, such as in teeth with short clinical crowns and extensive destruction of the tooth structure [7]. Additionally, endocrowns are indicated for teeth with curved, calcified, short, or narrow root canals or fractured instruments in the canal that prevent the application of a post [2,3,7].

Although endocrown restorations have been proven effective in molars, there is ongoing debate regarding their use in premolars and anteriors [9,12]. In the case of premolars, several researchers have reported a higher failure rate for premolar endocrowns compared to molars [7,10]. This is attributed to the smaller dimensions of the pulp chamber, resulting in a reduced bonding surface area [3,7]. Additionally, the greater ratio of preparation to the overall crown height in premolars creates a higher leverage effect that decreases fracture resistance, particularly when non-axial forces are received [3,7,11]. Premolars are more susceptible to these non-axial forces [3,11]. However, some recent studies have suggested that premolars exhibit similar clinical performance to molars [12].

As for anteriors, which are similar to premolars, they have a limited bonding surface area and receive higher non-axial forces [3,9,11]. Limited studies have been conducted in this area, but those that have been done indicate that stress in anterior endocrowns is higher compared to conventional post and core restorations and crowns [10]. This is because of the increased non-axial forces that anteriors are subjected to [3,11]. The lack of studies and conflicting findings present challenges to arriving at definitive conclusions regarding the use of endocrowns for anterior teeth [10].

In limited recent studies, researchers have examined the use of endocrowns as abutments for fixed partial dentures [13-15]. The findings indicate that endocrown abutments can be a viable alternative to conventional crown abutments [13-15].

Contraindications

Endocrowns are contraindicated in cases where there is extensive loss of tooth structure beneath the cementoenamel junction, preventing proper adhesion, or when the pulp chamber is shallow [2,3,12]. There are no established guidelines that specify the appropriate depth of the pulp chamber [3]. Nevertheless, certain studies indicated that a depth of at least 2 mm was necessary to ensure sufficient stabilization [3,12]. Endocrowns are also contraindicated in cases of parafunctional habits that increase lateral stress, as indicated by steep occlusal anatomy, wear, or facets [2,3].

Preparation

Endocrown restorations achieve macromechanical retention through anchorage to the pulp chamber and cavity margins [7]. This minimally invasive bonded restoration requires a specific preparation technique that differs from traditional crowns [8,16]. However, some modifications can be made in the preparation to compensate for aesthetic, biomechanical, or different material requirements [1,16].

Occlusal and External Axial Wall Preparation

For ceramic materials, a minimum occlusal reduction of 2 mm is recommended, whereas a reduction of 1-1.5 mm is sufficient for composite materials [3,8]. This is because of the elasticity and stress-absorption properties of composites [3]. The thickness of the ceramic restoration is measured from the margins of the axial wall to the maximum occlusal limit, typically ranging from 3 to 7 mm [1,7,8]. Several researchers have reported an increase in fracture resistance with greater thickness [7,8,11].

To achieve occlusal reduction, one approach is to drill 2 mm guide grooves, followed by a wheel diamond bur to reduce the occlusal surface along the long axis of the tooth and parallel to the occlusal plane [2,8]. The bur helps maintain proper alignment and creates a flat surface, resulting in a butt joint margin [2,8].

A butt joint, also known as a cervical sidewalk, refers to a 90-degree circumferential band of enamel margin with a width of 1 to 2 mm [3,5]. This type of margin enhances bonding and provides a stable surface that can withstand compressive stresses [2,5,8,17].

Ferrule Effect

Endocrown preparations typically do not involve the use of a ferrule [10,11]. However, there is an alternative design option for endocrown preparation that incorporates the ferrule effect along with a shoulder finish line [9,17,18]. The ferrule effect refers to a collar encircling the dentin’s parallel walls, extending in a 360-degree manner above the preparation’s shoulder [8]. In this design, it follows the same concept as the butt joint, but with the addition of a 90-degree shoulder margin positioned on the vertical wall [18]. This margin has a width of 1 mm and is located in the sound enamel [18]. Its purpose is to provide extra-short axial walls that counteract shear stress, resulting in improved marginal load control and better load distribution in the pulpal floor [17].

When comparing the butt joint design to the shoulder design with a ferrule, the butt joint design is less complex and has superior marginal integrity and internal adaptation [8,18]. However, studies of the effects of the ferrule and shoulder design have yielded conflicting results [1,10]. Some researchers suggest that the shoulder design with a ferrule offers greater fracture resistance and a lower incidence of catastrophic failures compared to the butt joint design [1,8-10,17]. Others have reported no significant differences in stress distribution and fracture resistance between the two designs [1].

For optimal outcomes, it is ideal to maintain the margins supragingivally in a circumferential manner [8,10]. Additionally, any undermined enamel should be removed [8].

Pulp Chamber Preparation

The preparation of the pulp chamber involves removing the undercuts in the access cavity using a cylindrical conical diamond bur with a 7-degree occlusal taper, which creates a continuous chamber and access cavity [2,8]. The bur should be held parallel to the long access of the tooth without touching the pulp chamber to create smooth, tapered walls [8]. However, excessive pressure should be avoided because it will reduce much of the wall thickness [8].

Regarding the depth of the pulp chamber, several researchers reported that trying to increase the pulp chamber depth did not affect fracture resistance, but led to more catastrophic failure [1,10,18]. When it comes to the chamber floor, it is advisable to remove the gutta-percha up to a depth of 2 mm to obtain a saddle anatomy of the floor, which provides more stability [3]. However, researchers have reported that extension into canals can reduce stress distribution properties and result in decreased marginal and internal adaptation [1,10].

Moreover, some researchers recommend using immediate dentin sealing with a bonding agent immediately after preparation to improve adhesion and reduce microleakage [3,5]. Additionally, they recommend filling irregularities in the pulp chamber with resin composite to eliminate retentive areas and prevent sliding or adjustment of the restoration [3]. However, other researchers have shown that neither immediate sealing nor the use of composites contributes to improved fracture resistance [10].

**Figure 1:**
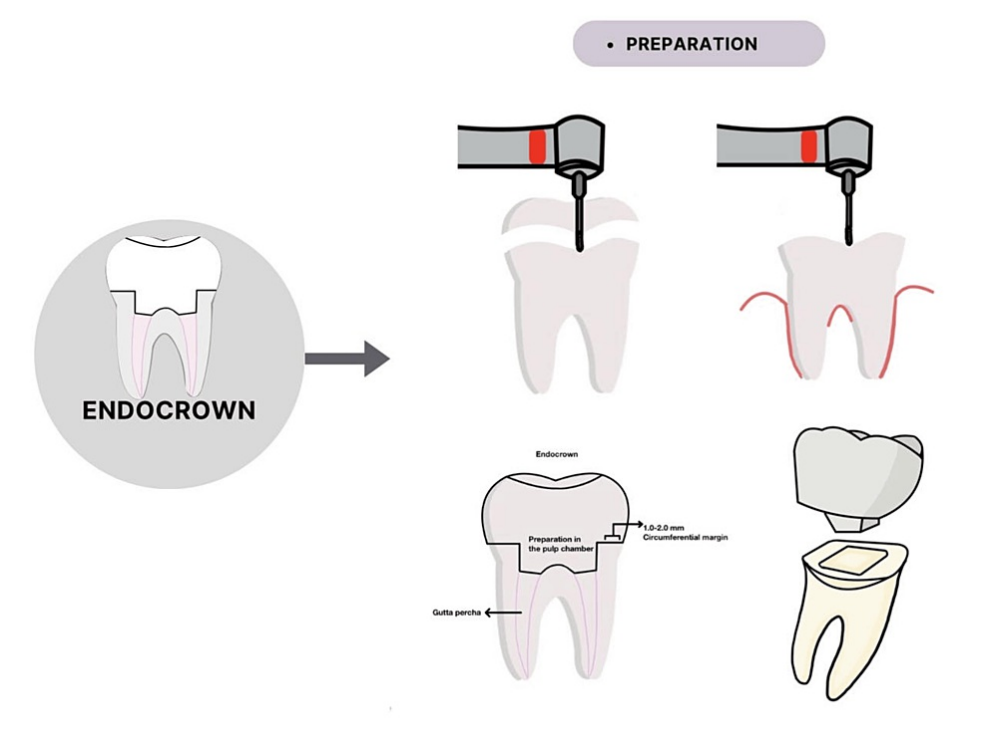
Brief view of endocrown preparation

Manufacturing

Two methods can be utilized for fabricating endocrowns: heat pressing or CAD/CAM [19]. In the heat pressing technique, the endocrown is manufactured in a dental laboratory using a cast derived from a conventional impression [19]. Polyether or polyvinyl siloxane is frequently used for the impression because of their perfect stability [19]. Once the cast is created, a wax pattern is produced and subsequently burned out to form a refractory mold [20]. Finally, ceramic ingots are heat-pressed in a ceramic furnace and inserted into the refractory mold to produce the restoration [20]. The technique offers several advantages, including its simplicity, time efficiency, familiarity among laboratory technicians, and ability to provide an appropriate fit for the restoration [21].

In the CAD/CAM procedure, a digital impression is taken using a scanner [22]. This can be done either by directly scanning the prepared tooth intraorally or by scanning the cast made from a conventional impression [20]. The restoration is then designed using CAD software. The subsequent CAM process involves milling a ceramic block to create the final restoration [20,22]. The utilization of CAD/CAM technology offers numerous advantages. It enables the production of high-quality and aesthetically pleasing restorations in a single session [2,7,22]. It allows for the selection of anatomical features that adapt to the scanned preparation and the opposing tooth, eliminating the need for diagnostic wax [2]. Additionally, data can be saved and easily tracked within the device, thereby saving storage space [22].

When comparing the marginal and internal fit of endocrowns produced using heat pressing and CAD/CAM techniques, studies have demonstrated that the CAD/CAM production method yields superior marginal and internal adaptation compared to heat pressing [19,23-25]. However, it is important to note that both techniques still exhibit clinically acceptable levels of marginal and internal discrepancy [23,24].

Materials

Several materials, such as lithium disilicate glass-ceramic, zirconia-reinforced lithium silicate glass-ceramic, zirconia, and resin composites, have been used to fabricate endocrowns [1,24,26]. The choice of material can impact the mechanical properties and the performance of the endocrown [24].

Lithium disilicate glass-ceramic is highly recommended because of its favorable mechanical properties, aesthetic outcomes, and the ability to bond to the tooth structure [21,24,27]. Additionally, studies have shown that it exhibits the highest fracture resistance compared to other materials, especially under lateral loading [1,24,27,28].

Zirconia-reinforced lithium silicate glass-ceramic is a glass-based ceramic material that incorporates zirconia particles into its matrix to enhance its mechanical and physical properties [21,25]. However, although the addition of zirconia particles increases the material’s strength and resistance to deformation, it also reduces the bonding strength between the restoration and tooth structure [24,28,29]. Additionally, the high modulus of elasticity of this material concentrates stress on the weakest points, potentially leading to catastrophic tooth failure [10,28,29].

Zirconia, a polycrystalline ceramic material devoid of glass phases, is widely used because of its excellent mechanical properties, making it suitable for high-stress situations like bruxism [30,31]. However, zirconia lacks the ability to be etched using routine methods, which can result in low bond strength and the potential debonding of the restoration [25,30]. Furthermore, it has exhibited the highest rate of catastrophic failures among other materials [1,10,30].

Resin composite materials have been introduced for endocrown fabrication as an alternative to ceramic materials, primarily because of their low elastic modulus, which is similar to dentin [10,24]. This similarity enables proper stress distribution, resulting in more favorable modes of failure [7,26,28]. Additionally, unlike ceramics, resin composites can be adjusted and repaired intraorally [12]. Furthermore, some researchers have reported that resin composites exhibit the highest fracture resistance compared to other materials [1,7,10]. However, it is worth noting that these materials tend to have a higher degree of marginal leakage [1,7,10].

Cementation

Adhesive cement, which plays a main role in the endocrown’s performance and durability, help an endocrown obtain micromechanical retention [3,7]. Adhesion helps distribute stress effectively, resulting in increased fracture resistance [11]. Additionally, if adhesion is lost, it can lead to issues like microleakage, secondary caries, and periodontal problems [32].

Resin cements are commonly used for endocrown cementation because of their excellent bonding strength, aesthetic features, high mechanical properties, and low solubility [7,33]. They can be classified as conventional resin cements or self-adhesive cements [33]. Conventional resin cements require multiple bonding steps, including surface treatment of both the tooth structure and the restoration using etchants and bonding agents [32,34]. These steps prolong the operation time and increase the risk of contamination, making the procedure more technique-sensitive [32,34]. To simplify the process and eliminate the need for surface treatment, self-adhesive resin cements have been developed [32,34]. However, some researchers have reported that the bond strength of self-adhesive resin cements is lower compared to conventional resin cements [32,34].

Resin cements can be categorized based on their polymerization method: self-cured, light-cured, and dual-cured [33]. Self-cured resin cements have limited applications because of their lower mechanical and aesthetic properties and shorter working times [33]. Light-cured resin cements offer an extended working time but are suitable only for shallow preparations [33]. This is because the light may be obstructed when it reaches deeper areas, potentially leading to adhesive failure [12,33]. Dual-cured resin cements have the advantage of being both self-cured and light-cured, making them useful for deep cavities [33]. They exhibit excellent mechanical properties and provide an extended working time [33]. The polymerization of resin cement can be hindered by remnants of eugenol-containing root canal sealers [7]. This issue can be addressed by cleaning and acid-etching the walls [7]. After cementation, it is important to remove any excess cement, especially in subgingival margins [27]. Radiographs can be taken to ensure there is no residual cement [27].

## Conclusions

Nowadays, endocrowns are a highly recommended choice for restoring teeth that have undergone endodontic treatment. In comparison to post-core and crowns, endocrowns are less invasive and offer a restoration that is aesthetically pleasing, provides sufficient retention, and possesses excellent mechanical properties. Although the use of endocrowns is commonly recommended for molars, further research is needed to determine their suitability for premolars and anterior teeth. Among the various materials available for fabricating endocrowns, lithium disilicate is the most frequently recommended option. Finally, it is crucial to emphasize that the success of the treatment relies on careful case selection and a thorough implementation of the adhesive procedure.
